# Impact of initiation of amikacin liposome inhalation suspension on hospitalizations and other healthcare resource utilization measures: a retrospective cohort study in real-world settings

**DOI:** 10.1186/s12890-022-02257-8

**Published:** 2022-12-03

**Authors:** Timothy Aksamit, Jasmanda Wu, Mariam Hassan, Emily Achter, Anjan Chatterjee

**Affiliations:** 1 Pulmonary Disease and Critical Care Medicine, Mayo Clinic, Rochester, MN USA; 2 Insmed Incorporated, Bridgewater, NJ USA; 3 STATinMED Research, Plano, TX USA

**Keywords:** Liposomal amikacin, ALIS, Claims-data analysis, Healthcare resource utilization, Hospitalization, *Mycobacterium avium*, Nontuberculous mycobacterial lung disease

## Abstract

**Background:**

*Mycobacterium avium* complex lung disease (MAC-LD) is an infection that is increasing in frequency, associated with substantial disease burden, and often refractory to treatment. Amikacin liposome inhalation suspension (ALIS) is the first therapy approved for refractory MAC-LD. In the CONVERT study of adult patients with refractory MAC-LD, adding ALIS to a multidrug background regimen showed evidence of MAC infection elimination in sputum by month 6, which was maintained in most patients through the end of treatment (≤ 12 months post-conversion). This study assessed changes in healthcare resource utilization (HCRU) among patients initiating ALIS in real-world settings.

**Methods:**

This retrospective cohort study of the All-Payer Claims Database (October 2018–April 2020) included patients aged ≥ 18 years with ≥ 1 pharmacy claim for ALIS and ≥ 12 months of continuous health plan enrollment pre- and post-ALIS initiation. Respiratory disease-related (and all-cause) HCRU (hospitalizations, length of stay [LOS], emergency department [ED] visits, and outpatient office visits) were compared 12 months pre- and post-ALIS initiation. Outcomes were reported at 6-month intervals; 0–6 months pre-ALIS initiation was the reference period for statistical comparisons.

**Results:**

A total of 331 patients received ALIS, with HCRU highest in the 6 months pre-ALIS initiation. Compared with 26.9% during the reference period, respiratory-related hospitalizations decreased to 19.3% (*P* < 0.01) and 15.4% (*P* < 0.0001) during 0–6 and 7–12 months post-ALIS initiation, respectively. Mean number of respiratory disease-related hospitalizations per patient/6-month period decreased from 1.0 (reference period) to 0.6 (*P* < 0.0005) at both timepoints post-ALIS initiation. A similar pattern was observed for all-cause hospitalizations and hospitalizations per patient/6-month period (both *P* < 0.005). Reductions in all-cause and respiratory disease–related LOS post-ALIS initiation were significant (both *P* < 0.05). ED visits were few and unchanged during the study. Significant reductions per patient/6-month period in all-cause and respiratory-related outpatient office visits were observed post-ALIS initiation (all *P* < 0.01).

**Conclusions:**

In this first real-world study of ALIS, respiratory disease-related (and all-cause) hospitalizations and outpatient visits were reduced in the 12 months following ALIS initiation. The results of this study provide HCRU-related information to better understand the impact of initiating ALIS treatment.

***Trial registration*:**

Not appliable.

**Supplementary Information:**

The online version contains supplementary material available at 10.1186/s12890-022-02257-8.

## Background

Nontuberculous mycobacterial lung disease (NTM-LD) is often a chronic, progressive, and debilitating disease [1, 2]. Population-based studies have shown that patients with NTM-LD have a higher associated burden of illness, including increased rates of hospitalization and mortality, than those without NTM-LD [3–6]. In the United States (US), the estimated prevalence of NTM-LD has risen from 6.8 per 100,000 persons in 2008 to 11.7 per 100,000 in 2015 [7]. This rise in prevalence may be due to a variety of factors, including aging populations, the ubiquitous use of immunosuppressive therapies and steroids, improved diagnostics, and increasing environmental prevalence [6–9].

*Mycobacterium avium* complex (MAC) accounts for 55 − 85% of NTM-LD cases in the US [8–12] and is the greatest contributor to the increase in NTM-LD prevalence [13]. A recent meta-analysis of 14 studies from Asia, Europe, and North America estimated the 5-year all-cause mortality for MAC-LD was 27% [14]. Failure to respond to treatment is common, and approximately 20–40% of MAC-LD cases are refractory to treatment [15–18]. Refractory MAC-LD is associated with increased healthcare resource utilization (HCRU) [19] and a reported 2-year mortality of 45% [20]. MAC-LD is difficult to treat due to the need for prolonged multidrug regimens, issues with regimen tolerability, drug resistance, and high rates of treatment failure [1, 15, 21, 22]. The current recommendation for guideline-based treatment (GBT) for initial treatment of MAC-LD is a three-drug combination of a macrolide plus ethambutol with a third antibiotic (e.g., rifamycin), all taken for at least 12 months after culture conversion (with intensification in patients with refractory disease) [23]. Collectively, treatment challenges and high morbidity and mortality rates indicate a substantial unmet need for more effective management of MAC-LD.

Amikacin liposome inhalation suspension (ALIS) is the first therapy indicated for the treatment of adults with refractory MAC-LD as part of a combination antibacterial drug regimen [24]. In the phase 3 CONVERT study (NCT02344004), addition of ALIS to a multidrug background regimen (antimicrobial regimen of ≥ 2 antibiotics) [25] in patients with refractory MAC-LD demonstrated a significantly higher rate of sputum conversion by month 6: 29% for ALIS plus background regimen versus 8.9% for background regimen alone (adjusted odds ratio, 4.22; 95% confidence interval [CI], 2.08–8.57; *P* < 0.001) [26]. The 12-month open-label extension study for CONVERT (NCT02628600) included patients with up to 20 months of treatment with ALIS [27]. Of the patients who achieved culture conversion by month 6, 55.4% of converters (36/65) in the ALIS plus GBT treated arm versus no converters (0/10) in the GBT alone arm achieved sustained and durable conversion (*P* = 0.0017) [24, 27]. In this extension study, culture conversion was observed at time points beyond 6 months of treatment, supporting the potential benefit of extended ALIS use in patients considered refractory to initial treatment [26]. In 2018, ALIS became the first therapy to receive accelerated approval by the Food and Drug Administration (FDA) for adult patients with refractory MAC-LD who have limited or no alternative treatment options. International treatment guidelines updated in 2020 recommend the addition of ALIS in patients with MAC-LD who have failed therapy after at least 6 months of GBT [23].

While ALIS provides an approved treatment option for refractory MAC-LD, the potential of ALIS to reduce economic burden in terms of HCRU is also of interest. However, to our knowledge, there are no real-world data available for ALIS to date. The objective of this study was to assess changes in hospitalization and other HCRU outcomes among patients before and after initiation of ALIS in the real-world setting.

## Methods

### Study design and data source

This retrospective, non-interventional, observational cohort study utilized the All-Payer Claims Database (APCD). Data in the APCD are sourced directly from claims clearing houses responsible for managing claims transactions for Medicaid, Medicare Fee-for-Service, Medicare Advantage, and commercial plans and include more than 300 million unique patients across all US geographic locations, and it is geographically representative of the US population. The APCD is 100% compliant with guidelines of the Health Insurance Portability and Accountability Act [27].

This study was performed in accordance with relevant guidelines and regulations. Since the study did not involve the collection, use, or transmittal of individually identifiable data, Institutional Review Board (IRB) approval was not required. This study analyzed only de-identified data which are priori exempt from the Federal Policy for the Protection of Human Subjects (1991) and does not meet the identification criteria necessary to be privileged under HIPAA [28]. As such, IRB approval to conduct this study was not required and considered exempt according to 45CFR46.101(b) (4): Existing Data and Specimens—No Identifiers. Informed consent to participate in this retrospective study was optional but not necessary under HIPAA; the process for obtaining consent was at the discretion of the healthcare provider [28]. The APCD is owned by STATinMED and no further permission to access the database was required.

### Cohort identification

We identified patients with at least 1 pharmacy claim for ALIS (identified using National Drug Code 71558-0590-28) between October 2018 and April 2020 (cohort identification period). The date of first ALIS prescription was defined as the index date. On the index date, patients were required to be at least 18 years of age and continuously enrolled in a health plan for 12 months before and after this date (the complete study period was October 2017 to April 2021). The outcomes before and after ALIS initiation for the same cohort of patients were compared and each patient served as its own control. In the absence of an International Classification of Diseases, Tenth Revision, Clinical Modification (ICD-10-CM) diagnostic code specific to MAC-LD, we aimed to include only ALIS prescriptions for treatment of refractory MAC-LD, as per the FDA–approved label; therefore, only ALIS pharmacy claims with an “adjudicated and approved” status by health plans/payers were allowed. As prior authorization submitted by service providers or patients to health plans/payers was required for obtaining approval to receive ALIS, health plans/payers would normally approve payments to pharmacy claims that were consistent with the ALIS coverage requirements (i.e., refractory MAC-LD) through the adjudication process.

### Baseline characteristics

Baseline demographic variables at index date included age, age category (18–24, 25–34, 35–44, 45–54, 55–64, 65–74, 75 + years), and sex. Additional patient characteristics at index date included health insurance (Medicare, Medicaid, and commercial plans) and US geographic region (Additional file 1: Table S1). Data on race were not captured. The modified Charlson Comorbidity Index (CCI) [29, 30], as well as clinical comorbidities (non-pulmonary and pulmonary comorbidities and symptoms) [31] were identified 0 − 12 months prior to ALIS initiation using ICD-10-CM codes (Additional file 2: Table S2). Concomitant antibiotics/antibiotic classes (Additional file 3: Table S3) were also identified 0–12 months prior to ALIS initiation.

### Study outcomes

The primary HCRU outcome of interest was hospitalization, which included inpatient stays including hospital emergency department (ED) visits that led to inpatient admission. All-cause hospitalization was defined as hospitalization for any reason. Respiratory disease–related hospitalization was based on an ICD-10-CM diagnostic code specific to respiratory disease diagnoses (J00–J99) being listed in position 1 and/or 2 on inpatient claims.

Additional HCRU outcomes included ED visits that occurred in the outpatient setting only (e.g., did not lead to an inpatient stay) and outpatient office visits. Outpatient office visits included ambulatory visits such as outpatient hospital visits and mobile ambulatory, urgent care, and telehealth visits (providers included primary care, infectious disease, and pulmonology physicians). All-cause ED or outpatient office visits were defined as visits for any reason. Respiratory disease–related ED or outpatient office visits were based on ICD-10-CM diagnostic codes specific to respiratory disease diagnosis being listed in any position of the associated claims.

HCRU outcomes were reported for every 6-month interval of the study period as this is a typical time frame for patient evaluation in clinical practice and was consistent with the interval used in the CONVERT study [26]. The baseline period encompassed 12 months (7–12 and 0–6 months) pre-ALIS initiation. The follow-up period encompassed 12 months (0–6 and 7–12 months) post-ALIS initiation.

Treatment patterns for ALIS and other antibiotics, including those commonly used in GBT, were reported. Patients with a ≥ 60-day lapse after their final ALIS prescription were considered to have discontinued ALIS treatment, with proportions of these patients described at 0–3 months, 4–6 months, 7–9 months, and 10–12 months. Use of selected other antibiotics was described before and after initiation of ALIS.

### Statistical analysis

Patient demographics, comorbidities (diseases and symptoms), antibiotic use, and all HCRU outcomes were summarized using descriptive statistics. Mean and standard deviation (SD) were used to summarize continuous variables and *n* (%) was used to summarize categorical variables. For HCRU outcomes, a period of 0–6 months prior to ALIS initiation served as the reference period for all statistical comparisons. The number and proportion of patients with each HCRU outcome, including the mean ± SD HCRU per patient per 6-month period, were reported.

To account for the paired nature of this analysis, McNemar’s Χ^2^ tests were used to compare the proportion of patients with an HCRU outcome (hospitalization, ED visit, outpatient office visit) in the reference period (0–6 months before ALIS initiation) to the proportion of patients in each 6-month follow-up period (0–6 and 7–12 months post-ALIS initiation). Wilcoxon signed rank tests were used to compare the number of HCRU outcomes per patient per 6-month period and length of stay per hospital admission in the reference period versus each follow-up period. *P* < 0.05 was considered statistically significant; no multiple comparisons adjustment was conducted. Data analyses were conducted using SAS statistical software version 9.4 (Cary, NC).

## Results

### Demographics and baseline clinical characteristics

**Fig. 1:**
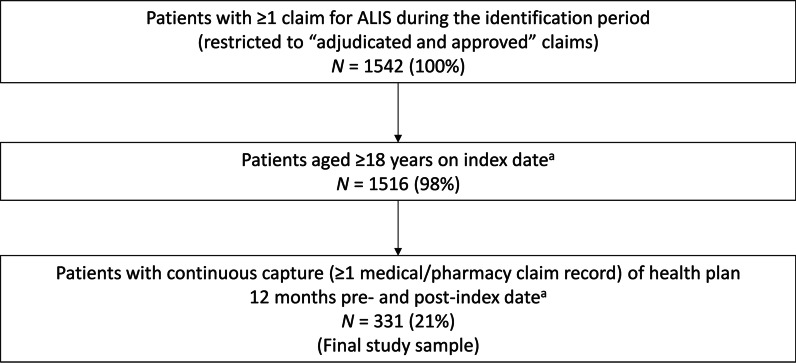
ALIS cohort identification and patient attrition. The outcomes before and after ALIS initiation for the same patient population were compared. *ALIS* amikacin liposome inhalation suspension. ^a^Index date was defined as the date of the first ALIS prescription

A total of 1542 patients who had at least one “adjudicated and approved” pharmacy claim for ALIS were identified (Fig. 1). Of these, 331 were aged ≥ 18 years with a continuous health plan during the 12 months pre- and post-ALIS initiation and formed the analysis population (Fig. 1). The outcomes before and after ALIS initiation for the same patient population were compared.

Patient demographics at index date as well as comorbidities and antibiotic use during the pre-ALIS baseline period are shown in Tables 1 and 2. Patients were predominantly female (77.9%) and aged ≥ 55 years (84.0%), with 65.9% enrolled in Medicare. Most patients (79.5%) had medical claims for NTM-LD. Bronchiectasis (57.1%) and chronic obstructive pulmonary disease (COPD; 45.6%) were the most common pulmonary comorbidities, and cardiovascular disease (58.9%) and hypertension (41.7%) were the most common non-pulmonary comorbidities. The common concomitant antibiotics used during the 12-month period prior to initiating ALIS were macrolides (40.2%), fluoroquinolones (29.0%) and rifamycin (26.3%), with 22.4% using a GBT three-drug regimen.

**Table 1:** Demographic and clinical characteristics of patients during the 12 months prior to initiating ALIS

	ALIS cohort (*N* = 331)
Age, mean (SD), years	64.6 (16.0)
*Age categories, n (%)*	
18 − 24 years	17 (5.1)
25 − 34 years	10 (3.0)
35 − 44 years	12 (3.6)
45 − 54 years	14 (4.2)
55 − 64 years	71 (21.5)
65 − 74 years	115 (34.7)
75 + years	92 (27.8)
*Sex, n (%)*	
Female	258 (77.9)
Male	73 (22.1)
*Health insurance, n (%)*	
Commercial	92 (27.8)
Medicare	218 (65.9)
Medicaid	21 (6.3)
*US geographic region, n (%)*	
Northeast	74 (22.4)
North Central	42 (12.7)
South	152 (45.9)
West	63 (19.0)
CCI, mean (SD)	2.2 (2.0)
Smoking history, n (%)	64 (19.3)
*Pulmonary comorbidities/symptoms, n (%)*	
NTM-LD	263 (79.5)
Other pulmonary comorbidities and symptoms	311 (94.0)
Bronchiectasis	189 (57.1)
COPD	151 (45.6)
Dyspnea	132 (39.9)
Cough	125 (37.8)
Asthma	74 (22.4)
Emphysema	51 (15.4)
Hemoptysis	44 (13.3)
Cystic fibrosis with pulmonary manifestations	33 (10.0)
Aspergillosis	28 (8.5)
Idiopathic pulmonary fibrosis	27 (8.2)
Idiopathic interstitial lung disease	19 (5.7)
Malignant neoplasm of bronchus and lung	15 (4.5)
Simple and mucopurulent chronic bronchitis	15 (4.5)
Diffuse panbronchiolitis	8 (2.4)
Pulmonary tuberculosis	7 (2.1)
Lung transplant	6 (1.8)
Pneumonia	2 (0.6)
Non-pulmonary comorbidities, *n* (%)	274 (82.8)
Cardiovascular disease	195 (58.9)
Hypertension	138 (41.7)
Gastroesophageal reflux	97 (29.3)
Osteoporosis	60 (18.1)
Diabetes mellitus	59 (17.8)
Other cancers	42 (12.7)
Underweight	39 (11.8)
Malnutrition	38 (11.5)
Chronic kidney disease	30 (9.1)
Other NTM disease^a^	24 (7.3)
Chronic heart failure	15 (4.5)
Overweight and obesity	11 (3.3)
Rheumatoid arthritis	10 (3.0)
Transplant of kidney or liver	7 (2.1)
Sjögren syndrome	5 (1.5)
HIV/AIDS	2 (0.6)
Dementia	1 (0.3)

*ALIS* amikacin liposome inhalation suspension, *CCI* Charlson Comorbidity Index, *COPD* chronic obstructive pulmonary disease, *DMAC* disseminated mycobacterium avium-intracellulare complex, *HIV/AIDS* human immunodeficiency syndrome virus/acquired immunodeficiency syndrome, *NTM* nontuberculous mycobacterial, *NTM-LD* nontuberculous mycobacterial lung disease; *TB* tuberculosis

^a^Other NTM disease included cutaneous mycobacterial infection and DMAC

**Table 2:** Concomitant antibiotic use over the period of 12 months prior to initiating ALIS

	ALIS cohort
Antibiotic class/antibiotic	(*N* = 331)
GBT regimen^a^	74 (22.4)
Macrolide	133 (40.2)
Azithromycin	127 (38.4)
Clarithromycin	16 (4.8)
Ethambutol	83 (25.1)
Rifamycin	87 (26.3)
Rifabutin	13 (3.9)
Rifampin	82 (24.8)
Aminoglycoside	47 (14.2)
Amikacin sulfate	39 (11.8)
Tobramycin	14 (4.2)
Fluoroquinolone	96 (29.0)
Ciprofloxacin	50 (15.1)
Levofloxacin	61 (18.4)
Moxifloxacin	9 (2.7)
Other antibiotics classes	
Cephalosporin	6 (1.8)
Carbapenem	8 (2.4)
Oxazolidinone	14 (4.2)
Glycylcycline	5 (1.5)
Tetracycline	37 (11.2)

All data are presented as *n* (%)

*ALIS* amikacin liposome inhalation suspension, *GBT* guideline-based treatment

^a^GBT based on American Thoracic Society/European Respiratory Society/European Society of Clinical Microbiology and Infectious Diseases/Infectious Diseases Society of America clinical practice guidelines [23] guidelines refer to three-drug combination of a macrolide plus ethambutol with a third antibiotic, typically a rifamycin

### HCRU

#### Hospitalizations

**Fig. 2:**
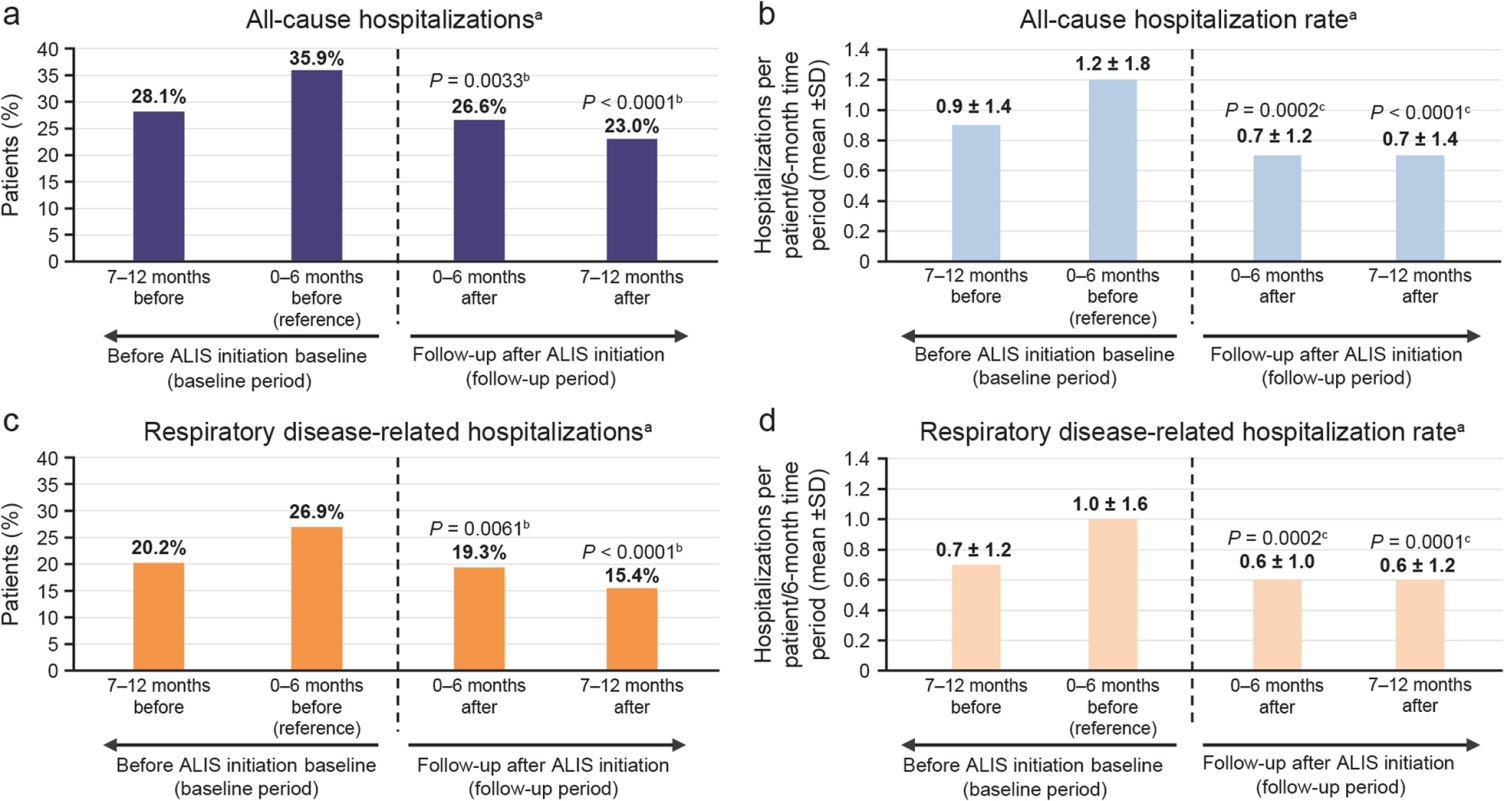
All-cause and respiratory-related hospitalizations. Proportion of patients with **a** all-cause and **c** respiratory disease–related hospitalizations. Hospitalization rate per 6 months pre- and post-ALIS initiation for **b** all-cause and **d** respiratory disease-related hospitalizations (*N* = 331). The outcomes before and after ALIS initiation for the same patient population were compared. *ALIS* amikacin liposome inhalation suspension, ED emergency department. ^a^Hospitalizations include inpatient stays as well as hospital ED visits that lead to inpatient admission. ^b^McNemar’s Χ^2^ test. ^c^Wilcoxon signed rank test

The proportion of patients with an all-cause hospitalization was 28.1% during the 7–12 months prior to ALIS initiation. In the 0–6 months prior to ALIS initiation (reference period), the percentage of patients with all-cause hospitalizations increased to 35.9% (Table 3 and Fig. 2). Following this, the proportion of patients hospitalized for any cause decreased significantly from 35.9% to 26.6% (*P* = 0.0033) and 23.0% (*P* < 0.0001) at 0 − 6 months and 7–12 months post-ALIS initiation, respectively (Fig. 2a); compared with the reference period, this translates to approximately 25% and 36% fewer patients, respectively, with all-cause hospitalizations. A similar trend was observed for the number of hospitalizations: the mean ± SD number of all-cause hospitalizations per patient per 6 months decreased significantly from 1.2 ± 1.8 in the reference period to 0.7 ± 1.2 (*P* = 0.0002) and 0.7 ± 1.4 (*P* < 0.0001) at 0–6 and 7–12 months post-ALIS initiation, respectively (equivalent to a 42% decrease in mean all-cause hospitalizations at both timepoints following the start of ALIS treatment) (Fig. 2b).

**Table 3:** Healthcare resource utilization pre- and post-ALIS initiation (*N* = 331)

	Baseline (pre-ALIS)		Follow-up (post-ALIS initiation)			
	7−12 months	0−6 months (reference)	0−6 months	*P* value	7−12 months	*P* value
Hospitalizations^*a*^						
Proportion of patients with hospitalizations, *n* (%)						
All-cause	93 (28.1)	119 (35.9)	88 (26.6)	0.0033^b^	76 (23.0)	< 0.0001^b^
Respiratory disease–related	67 (20.2)	89 (26.9)	64 (19.3)	0.0061^b^	51 (15.4)	< 0.0001^b^
Number of hospitalizations per patient per 6 months, mean ± SD						
All-cause	0.9 ± 1.4	1.2 ± 1.8	0.7 ± 1.2	0.0002^c^	0.7 ± 1.4	< 0.0001^c^
Respiratory disease–related	0.7 ± 1.2	1.0 ± 1.6	0.6 ± 1.0	0.0002^c^	0.6 ± 1.2	0.0001^c^
LOS (per hospital admission), mean ± SD, days						
All-cause	4.7 ± 4.1	6.2 ± 4.9	3.9 ± 3.8	0.0004^c^	4.9 ± 5.6	0.0055^c^
Respiratory disease–related	4.9 ± 4.1	6.2 ± 4.5	4.4 ± 4.0	0.0116^c^	5.2 ± 5.7	0.0209^c^
ED visits						
Proportion of patients with ED visits, *n* (%)						
All-cause	41 (12.4)	39 (11.8)	37 (11.2)	0.7576^b^	29 (8.8)	0.1573^b^
Respiratory disease–related	15 (4.5)	17 (5.1)	10 (3.0)	0.0896^b^	18 (5.4)	0.8415^b^
Number of ED visits per patient per 6 months, mean ± SD						
All-cause	0.6 ± 1.1	0.6 ± 1.2	0.7 ± 1.7	0.3202^c^	0.5 ± 1.1	0.1455^c^
Respiratory disease–related	0.5 ± 1.2	0.5 ± 0.9	0.4 ± 1.3	0.3619^c^	0.5 ± 0.9	0.7945^c^
Outpatient office visits						
Proportion of patients with outpatient visits, *n* (%)						
All-cause	294 (88.8)	296 (89.4)	289 (87.3)	0.1779^b^	285 (86.1)	0.0934^b^
Respiratory disease–related	193 (58.3)	219 (66.2)	196 (59.2)	0.0088^b^	179 (54.1)	< 0.0001^b^
Number of outpatient visits per patient per 6 months, mean ± SD						
All-cause	6.2 ± 7.5	7.3 ± 8.6	7.4 ± 13.0	0.0071^c^	6.8 ± 12.8	0.0028^c^
Respiratory disease–related	2.4 ± 3.3	3.3 ± 5.0	3.0 ± 9.4	< 0.0001^c^	2.2 ± 3.5	< 0.0001^c^

The outcomes before and after ALIS initiation for the same patient population were compared

*ALIS* amikacin liposome inhalation suspension, *ED* emergency department, *LOS* length of stay, *SD* standard deviation

^a^Hospitalizations included inpatient stays as well as hospital ED visits that led to inpatient admission

^b^McNemar’s Χ^2^ tests were used to test statistically significant differences

^c^Wilcoxon signed rank tests were used to test statistically significant differences

Results for respiratory disease–related hospitalizations were similar. The proportion of patients hospitalized for respiratory disease–related causes decreased significantly from 26.9% in the reference period to 19.3% (*P* = 0.0061) and 15.4% (*P* < 0.0001) at 0−6 and 7−12 months post-ALIS initiation, respectively (Fig. 2c), which when compared with the reference period equates to approximately 28% and 43% fewer patients, respectively, with respiratory disease–related hospitalizations. The mean ± SD number of respiratory disease–related hospitalizations per patient per 6 months also decreased significantly from 1.0 ± 1.6 in the reference period to 0.6 ± 1.0 (*P* = 0.0002) and 0.6 ± 1.2 (*P* = 0.0001) at 0–6 months and 7–12 months post-ALIS initiation, respectively (equivalent to a 40% decrease in mean respiratory disease–related hospitalizations at both timepoints following the start of ALIS treatment) (Fig. 2d).

Mean ± SD LOS associated with all-cause hospitalization was significantly reduced from 6.2 ± 4.9 days (reference period) to 3.9 ± 3.8 days (*P* = 0.0004) and 4.9 ± 5.6 days (*P* = 0.0055) in the 0–6 months and 7–12 months post-ALIS initiation, respectively (Table 3). Similarly, LOS for respiratory disease–related hospitalization was significantly reduced from 6.2 ± 4.5 days (reference period) to 4.4 ± 4.0 days (*P* = 0.0116) and 5.2 ± 5.7 days (*P* = 0.0209) in the 0–6 months and 7–12 months after ALIS initiation, respectively (Table 3).

#### ED visits

ED utilization in the outpatient setting was low throughout the study, with less than 13% of patients having an all-cause ED visit and less than 6% having a respiratory disease–related ED visit during any of the study assessment periods (Table 3). For ED visits, there were no significant differences in all-cause or respiratory disease–related utilization measures pre- versus post-ALIS initiation.

#### Outpatient office visits

Nearly 90% of patients had at least one outpatient office visit in every 6-month interval of the study period. All-cause outpatient office visits decreased slightly after initiation of ALIS (differences not statistically significant); however, statistically significant changes were observed in the proportion of patients with respiratory disease–related outpatient office visits, which decreased from 66.2% in the reference period to 59.2% (*P* = 0.0088) and 54.1% (*P* < 0.0001) in the 0–6 and 7–12 months post-ALIS initiation, respectively (Table 3). The mean ± SD number of respiratory disease–related outpatient office visits per patient per 6 months decreased significantly from 3.3 to 5.0 in the 6 months prior to ALIS to 3.0 ± 9.4 (*P* < 0.0001) and 2.2 ± 3.5 (*P* < 0.0001) in the 0–6 months and 7–12 months after ALIS initiation, respectively (Table 3).

### Treatment patterns

The numbers of patients who discontinued ALIS treatment during the follow-up period were 108 (32.6%) at 0–3 months, 51 (15.4%) at 4–6 months, 48 (14.5%) at 7–9 months, and 17 (5.1%) at 10–12 months. A trend of reduced use of other antibiotics was observed after initiation of ALIS treatment. (Additional file 4: Table S4).

## Discussion

This is the first real-world study of ALIS, currently the only approved treatment as part of a combination antibacterial drug regimen for adult patients with refractory MAC-LD who have limited or no alternative treatment options. We observed significant reductions in both all-cause and respiratory disease-related hospitalizations and outpatient office visits within 6 months of ALIS initiation. These significant reductions in HCRU were maintained during the subsequent 6 months for a total follow-up period of 1 year after ALIS initiation.

HCRU measures were highest in the 6 months immediately preceding ALIS initiation, which suggests that patients may have experienced a deterioration in their clinical condition, which would be consistent with medical concerns resulting in initiation of ALIS. It is notable that the significant reductions in hospitalizations and outpatient office visits were observed during 0–6 and 7–12 months post-ALIS initiation. While a third of patients discontinued ALIS after 3 months, we found similar trends in reduced hospitalization when we examined patients with varying lengths of ALIS treatment (unpublished observations [JW, MH, AC]). In addition, we observed that ALIS use was not displaced by increased use of other antibiotics, as their use also decreased (Additional file 4: Table S4). Another finding is that only 24.4% of patients in our study had been prescribed GBT in the baseline period before ALIS initiation. While this finding is consistent with previous reports of low rates of GBT in patients with NTM-LD (approximately 13–28%) [32–34], it underscores the need to explore potential barriers to adoption and to develop strategies to enhance adherence to treatment guidelines. In addition, previous studies have demonstrated a marked incremental burden of NTM-LD in patients with underlying respiratory diseases [3, 35]. Whether the reductions in HCRU that were observed in the current study were directly related to treatment of NTM-LD or other underlying respiratory comorbidities is not clear; however, it is plausible that with better management of NTM-LD, there may be better overall management of respiratory illness.

Overall, the patient demographics observed in this real-world study were consistent with reported epidemiologic data for patient characteristics and MAC-LD distribution in the US [8–12]. Baseline demographics (mean age 65 years; 78% female) were comparable with those of refractory patients in a US NTM-LD registry (age 67 years; 82% female) [18]. COPD was more common in our study population (46%) than in the NTM-LD registry (< 20%) [18] but consistent with the rate of COPD in a recent US claims-based NTM-LD epidemiology study (53%) [7]. In addition, approximately half the patients in the registry had a history of smoking, which was higher than the smoking rates observed in our and other studies (< 20%) [7]; this difference may be due to underreporting in claims data, as diagnostic codes for smoking have low sensitivity [36]. Over half of our study population had respiratory comorbidities, consistent with prior epidemiological reports of patients with NTM-LD [6, 7].

Patients with refractory MAC-LD tend to have more frequent hospitalizations and poor quality of life [19, 37]. Because pulmonary symptoms and exacerbations caused by respiratory diseases and NTM-LD may overlap, it is difficult to determine whether HCRU is related to NTM-LD specifically. Thus, the broader category of the respiratory disease–related HCRU was presented as our main study findings. Nevertheless, we conducted post hoc analyses using NTM-LD–specific diagnostic codes (i.e., in position 1 or 2 for hospitalization claims and in any position for ED and outpatient office visit claims) to examine NTM-LD–related HCRU (Additional file 5: Table S5). A smaller number of NTM-LD–related HCRU events were identified in the claims data, as expected; however, the overall trends were generally similar to those for all-cause and respiratory disease–related HCRU.

Given the broad effects of the coronavirus disease 2019 (COVID-19) pandemic on HCRU in the US, we explored the impact of a COVID diagnosis on our study findings. We searched the literature investigating the broad pandemic effects on HCRU in the US. The rate of hospital admissions from the ED was initially stable and then increased during the first four months of 2020 [38]. The respiratory illness–related hospitalizations per month remained stable from January to July 2020 [39]. Taken together, these findings suggest that the significant reductions in hospitalizations, particularly respiratory disease–related hospitalizations, observed in our study post-ALIS may not be attributable to the COVID-19 pandemic alone. Regarding outpatient office visit-related HCRU, it was found that telehealth visits more than doubled between January and October 2020 [40]. In our study, telehealth visits were included in outpatient office visits, therefore, the significant reductions in outpatient office visits post-ALIS are unlikely due to the COVID-19 pandemic alone. These findings suggest that the significant reductions in hospitalizations, particularly respiratory disease–related hospitalizations, observed in our study following ALIS initiation may not be attributable to the COVID-19 pandemic alone.

### Strengths and limitations

The APCD provides comprehensive data inclusive of Medicaid, Medicare, and commercial insurance plans over a nationwide US sample. Strengths of claims data include large sample sizes over broad geographic regions (coverage of 93% of the US), the ability to conduct longitudinal follow-up in a real-world setting and minimized selection bias [7]. Another advantage of our study design is that by comparing HCRU in the period before and after ALIS initiation, each patient served as their own control, which mitigated the potential for patient-related confounding variables. The pre-/post-intervention design is an established method for evaluating the HCRU impact of an intervention [41–44]. Given the limited number of patients available for evaluation and the difficulties in identifying an appropriate control group, the self-controlled aspect of our study design eliminated all time invariant confounding [41] and was especially well suited for a newly introduced treatment for MAC-LD.

Study limitations include the use of pharmacy claims for dispensed ALIS prescriptions to identify the patients who initiated ALIS because there is no ICD-10-CM diagnostic code specific to MAC-LD. However, we addressed this by utilizing the time stamp on the pharmacy claims to remove rejected claims that were not consistent with the ALIS label indications. This approach enabled us to include the retained pharmacy claims that were “adjudicated and approved” (i.e., consistent with ALIS label). Thus, patients in this study were likely to have MAC-LD.

Claims data have inherent limitations [4, 7, 45] as they are collected for the purpose of payment and therefore lack the granularity of important clinical data. Thus, we could not determine the NTM species, rate of sputum conversion, changes in clinical signs and symptoms, cause of hospitalization, real-world drug usage, and reasons for treatment discontinuation. The study did not assess external factors, such as disease seasonality, nevertheless this was unlikely to have impacted the observed trends in HCRU as patients were initiated on ALIS at different points of time and seasons spread throughout the year. The corresponding observation period for outcomes was also spread throughout the year. We examined all-cause and respiratory disease–related HCRU in NTM-LD patients, recognizing that real-world coding practices may not routinely capture the contribution of NTM-LD to HCRU, because the overlap between NTM-LD–related and respiratory disease–related symptoms can make it difficult to discern the cause of a pulmonary exacerbation (e.g., some pulmonary exacerbations associated with NTM-LD may be coded as respiratory disease rather than as NTM-LD specifically).

Finally, HCRU were assessed over a period of 12 months post-ALIS initiation; treatment guidelines recommend continuation of therapy for at least 12 months after culture conversion [23]. Further research to assess HCRU beyond 12 months following ALIS initiation is warranted.

## Conclusions

This study provides the first real-world evidence where significant reductions in all-cause and respiratory disease–related hospitalizations and outpatient office visits were observed after initiating ALIS. With the known increased economic burden of refractory MAC-LD care on the healthcare system, the results of this study provide resource utilization-related information to better understand the impact of initiating ALIS treatment.

## Supplementary Information

**Additional file 1.** Definition of US regions.

**Additional file 2.** ICD-10-CM codes for comorbidities.

**Additional file 3.** Antibiotic drug classes and individual drugs.

**Additional file 4.** Proportion (%) of patients on select antibiotics/antibiotic classes before and after initiating ALIS.

**Additional file 5.** NTM-LD–related HCRU pre- and post-ALIS initiation.

## Data Availability

The datasets supporting the conclusions of this article are included within the article (and its additional files).

## Abbreviations

ALIS: Amikacin liposome inhalation suspension
APCD: All-Payer Claims Database
CCI: Charlson Comorbidity Index
CI: Confidence interval
COPD: Chronic obstructive pulmonary disease
COVID-19: Coronavirus disease 2019
ED: Emergency department
GBT: Guideline-based treatment
HCRU: Healthcare resource utilization
ICD-10-CM: International Classification of Diseases, Tenth Revision, Clinical Modification
LD: Lung disease
MAC: *Mycobacterium avium* complex
NTM: Nontuberculous mycobacterial
OLE: Open-label extension
SD: Standard deviation
US: United States
