# Supplementary material for: Impact of initiation of amikacin liposome inhalation suspension on hospitalizations and other healthcare resource utilization measures: a retrospective cohort study in real-world settings
Source: BMC Pulm Med. 2022 Dec 3;22:461. doi: 10.1186/s12890-022-02257-8 (PMC9719199; doi:10.1186/s12890-022-02257-8)
Supplement: Supplementary file 1 — Additional file 1. Definition of US regions. [file 12890_2022_2257_MOESM1_ESM.docx]

**Table S1** Definition of US regions

| **US region** | **State** |
| --- | --- |
| Northeast | CT, MA, ME, NH, RI, VT, NJ, NY, PA |
| North Central | IL, IN, MI, OH, WI, IA, KS, MN, MO, ND, NE, SD |
| South | DC, DE, FL, GA, MD, NC, SC, VA, WV, AL, KY, MS, TN, AR, LA, OK, TX |
| West | AZ, CO, ID, MT, NM, NV, UT, WY, AK, CA, HI, OR, WA |
| Other | Puerto Rico, Virgin Islands |
