# Supplementary material for: Impact of initiation of amikacin liposome inhalation suspension on hospitalizations and other healthcare resource utilization measures: a retrospective cohort study in real-world settings
Source: BMC Pulm Med. 2022 Dec 3;22:461. doi: 10.1186/s12890-022-02257-8 (PMC9719199; doi:10.1186/s12890-022-02257-8)
Supplement: Supplementary file 2 — Additional file 2. ICD-10-CM codes for comorbidities. [file 12890_2022_2257_MOESM2_ESM.docx]

**Table S2** ICD-10-CM codes for comorbidities

| **Clinical comorbidities** | **ICD-10-CM code(s)** |
| --- | --- |
| NTM-LD |  |
| Pulmonary mycobacterial infection | A31.0 |
| Other mycobacterial infections | A31.8 |
| Mycobacterial infection, unspecified | A31.9 |
| Other pulmonary comorbidities and symptoms |  |
| Aspergillosis | B44 |
| Asthma | J45 |
| Bronchiectasis | J47 |
| COPD | J40−J42, J44 |
| Cough | R05 |
| CF with pulmonary manifestations | E84.0 |
| Diffuse panbronchiolitis | J21.9 |
| Dyspnea | R06.0 |
| Emphysema | J43 |
| Hemoptysis | R04.2 |
| Idiopathic interstitial lung disease | J84 |
| Idiopathic pulmonary fibrosis | J84.1 |
| Lung transplant | Z94.2 |
| Malignant neoplasm of bronchus and lung | C34, C46, C78, C7A0.90 |
| Pneumonia | A02.22, A21.2, A22.1, A37, A42−A43, A48, B01−B59, J09.9X1, J10−J18 |
| Pulmonary TB | A15.0 |
| Simple and mucopurulent chronic bronchitis | J41 |
| Smoking history | Z87.891 |
| Non-pulmonary comorbidities |  |
| Cardiovascular disease | I00−I99 |
| Chronic heart failure | I50.22−I50.23, I50.32−I50.33, I50.42−I50.43, I50.812−I50.813 |
| Chronic kidney disease | N18 |
| Dementia | F0150−F0151, F0280−F0281, F0390−F0391, G3109, G3183 |
| Diabetes mellitus | E08−E11, E13 |
| Gastroesophageal reflux | K20.1, K21 |
| Hypertension | H35, H40, I10−I13, I15−I16, I27.0−I27.2, I67.4 |
| HIV/AIDS | B20−B22, B24, B97.35 |
| Malnutrition | E43, E44, E46 |
| Osteoporosis | M80−M18 |
| Other cancers | C00−C7A, C81−C96 |
| Overweight and obesity | E66 |
| Rheumatoid arthritis | M05 |
| Sjögren syndrome | M35.0 |
| Transplant |  |
| Kidney | Z94.0 |
| Heart | Z94.1 |
| Liver | Z94.4 |
| Underweight | R63.4, R63.6 |
| Other NTM disease |  |
| Cutaneous mycobacterial infection | A31.1 |
| DMAC | A31.2 |

*COPD* chronic obstructive pulmonary disease, *DMAC* disseminated *Mycobacterium avium*-*intracellulare* complex, *HIV/AIDS* human immunodeficiency syndrome virus/acquired immunodeficiency syndrome, *ICD-10-CM* International Classification of Diseases, Tenth Revision, Clinical Modification, *NTM* nontuberculous mycobacterial, *NTM-LD* nontuberculous mycobacterial lung disease, *TB* tuberculosis
