# Supplementary material for: Impact of initiation of amikacin liposome inhalation suspension on hospitalizations and other healthcare resource utilization measures: a retrospective cohort study in real-world settings
Source: BMC Pulm Med. 2022 Dec 3;22:461. doi: 10.1186/s12890-022-02257-8 (PMC9719199; doi:10.1186/s12890-022-02257-8)
Supplement: Supplementary file 3 — Additional file 3. Antibiotic drug classes and individual drugs. [file 12890_2022_2257_MOESM3_ESM.docx]

**Table S3** Antibiotic drug classes and individual drugs

| **Class** | **Drug name** | **Class** | **Drug name** |
| --- | --- | --- | --- |
| aminoglycoside | amikacin sulfate | cephalosporin | cefoxitin |
|  | gentamicin |  | ceftazidime |
|  | streptomycin |  | cefaclor |
|  | tobramycin |  | cefadroxil |
| fluoroquinolone | ciprofloxacin |  | cefazolin |
|  | gemifloxacin |  | cefdinir |
|  | levofloxacin |  | cefditoren |
|  | moxifloxacin |  | cefepime |
| carbapenem | imipenem |  | cefiderocol |
|  | doripenem |  | cefotaxime |
|  | ertapenem |  | cefotetan |
|  | meropenem |  | cefpodoxime |
| oxazolidinone | tedizolid |  | cefprozil |
|  | linezolid |  | ceftaroline |
| glycylcycline | tigecycline |  | ceftolozane |
| tetracycline | doxycycline |  | ceftriaxone |
|  | eravacycline |  | cefuroxime |
|  | demeclocycline |  | cephalexin |
|  | minocycline | macrolide | azithromycin |
|  | sarecycline |  | clarithromycin |
|  | tetracycline |  | erythromycin |
|  | tigecycline |  | fidaxomicin |
|  | omadacycline | ethambutol | ethambutol |
| riminophenazine | clofazimine | rifamycin | rifabutin |
| diarylquinoline | bedaquiline |  | rifampin |
