# Supplementary material for: Impact of initiation of amikacin liposome inhalation suspension on hospitalizations and other healthcare resource utilization measures: a retrospective cohort study in real-world settings
Source: BMC Pulm Med. 2022 Dec 3;22:461. doi: 10.1186/s12890-022-02257-8 (PMC9719199; doi:10.1186/s12890-022-02257-8)
Supplement: Supplementary file 4 — Additional file 4. Proportion (%) of patients on select antibiotics/antibiotic classes before and after initiating ALIS. [file 12890_2022_2257_MOESM4_ESM.docx]

**Table S4** Proportion (%) of patients on select antibiotics/antibiotic classes before and after initiating ALIS

| **Antibiotic/**  **antibiotic class** | **Baseline (pre-ALIS)** | | **Follow-up (post-ALIS initiation)** | |
| --- | --- | --- | --- | --- |
|  | **7**−**12 months** | **0**−**6 months (reference)** | **0**−**6 months** | **7**−**12 months** |
| Macrolides | 86 (26%) | 100 (30%) | 89 (27%) | 89 (27%) |
| Ethambutol | 44 (13%) | 62 (19%) | 57 (17%) | 50 (15%) |
| Rifamycins | 44 (13%) | 60 (18%) | 52 (16%) | 46 (14%) |
| Aminoglycosides^a^ | 17 (5%) | 36 (11%) | 13 (4%) | 12 (4%) |
| Fluoroquinolones | 51 (15%) | 47 (14%) | 25 (8%) | 30 (9%) |

*ALIS* amikacin liposome inhalation suspension

^a^Aminoglycosides, excluding ALIS
