# Supplementary material for: Impact of initiation of amikacin liposome inhalation suspension on hospitalizations and other healthcare resource utilization measures: a retrospective cohort study in real-world settings
Source: BMC Pulm Med. 2022 Dec 3;22:461. doi: 10.1186/s12890-022-02257-8 (PMC9719199; doi:10.1186/s12890-022-02257-8)
Supplement: Supplementary file 5 — Additional file 5. NTM-LD–related HCRU pre- and post-ALIS initiation. [file 12890_2022_2257_MOESM5_ESM.docx]

**Table S5** NTM-LD–related HCRU pre- and post-ALIS initiation

|  | **Baseline (pre-ALIS)** | | **Follow-up (post-ALIS initiation)** | | | |
| --- | --- | --- | --- | --- | --- | --- |
|  | **7**−**12 months** | **0**−**6 months (reference)** | **0**−**6 months** | ***P* value** | **7**−**12 months** | ***P* value** |
| Hospitalizations^a^ |  |  |  |  |  |  |
| Proportion of patients with hospitalizations, *n* (%) |  |  |  |  |  |  |
| NTM-LD–related | 28 (8.5) | 49 (14.8) | 40 (12.1) | 0.2164^b^ | 33 (10.0) | 0.0489^b^ |
| Number of hospitalizations per patient per 6 months, mean ± SD |  |  |  |  |  |  |
| NTM-LD–related | 0.4 ± 0.8 | 0.6 ± 0.9 | 0.5 ± 0.7 | 0.1981^c^ | 0.5 ± 1.3 | 0.3033^c^ |
| LOS (per hospital admission), days, mean ± SD |  |  |  |  |  |  |
| NTM-LD–related | 1.2 ± 3.4 | 1.8 ± 3.8 | 1.7 ± 4.2 | 0.5192^c^ | 2.2 ± 6.1 | 0.7798^c^ |

| ED visits |  |  |  |  |  |  |
| --- | --- | --- | --- | --- | --- | --- |

| Proportion of patients with ED visits, *n* (%) |  |  |  |  |  |  |
| --- | --- | --- | --- | --- | --- | --- |
| NTM-LD–related | 2 (0.6) | 5 (1.5) | 2 (0.6) | 0.1797^b^ | 2 (0.6) | 0.2568^b^ |
| Number of ED visits per patient per 6 months, mean ± SD |  |  |  |  |  |  |
| NTM-LD–related | 0.2 ± 0.4 | 0.5 ± 0.5 | 0.3 ± 0.7 | 0.7500^c^ | 0.2± 0.4 | 0.453^c^ |

| Outpatient office visits |  |  |  |  |  |  |
| --- | --- | --- | --- | --- | --- | --- |

| Proportion of patients with outpatient visits, *n* (%) |  |  |  |  |  |  |
| --- | --- | --- | --- | --- | --- | --- |
| NTM-LD–related | 128 (38.7) | 187 (56.5) | 179 (54.1) | 0.3096^b^ | 168 (50.8) | 0.0393^b^ |
| Number of outpatient visits per patient per 6 months, mean ± SD |  |  |  |  |  |  |
| NTM-LD–related | 1.4 ± 2.0 | 2.4 ± 2.5 | 2.4 ± 3.2 | 0.2536^c^ | 1.9 ± 2.3 | 0.0016^c^ |

*ALIS* amikacin liposome inhalation suspension, *ED* emergency department, *HCRU* healthcare resource utilization, *LOS* length of stay, *NTM-LD* nontuberculous mycobacterial lung disease, *SD* standard deviation

^a^Hospitalizations included inpatient stays as well as hospital ED visits that led to inpatient admission

^b^McNemar's Χ^2^ tests were used to test statistically significant differences

^c^Wilcoxon signed rank tests were used to test statistically significant differences
